# The dual role of baseline absolute eosinophil count in non-small cell lung cancer immunotherapy: a biomarker for enhanced efficacy and elevated risk of immune checkpoint inhibitor-related pneumonitis

**DOI:** 10.1186/s12885-026-16104-0

**Published:** 2026-04-27

**Authors:** Mengying Zhang, Ting Jiao, Yuwan Ma, Shuanying Yang

**Affiliations:** Department of Respiratory and Critical Care Medicine, The Second Affiliated Hospital of Xi’an Jiaotong University, Xi’an, 710004 China

**Keywords:** Non-small cell lung cancer, Immune checkpoint inhibitors, Eosinophils, Biomarker, Immune-related adverse events

## Abstract

**Objectives:**

This retrospective study aimed to evaluate the association between baseline absolute eosinophil count (AEC) and both survival outcomes and the risk of immune checkpoint inhibitor-related pneumonitis (ICI-pneumonitis) in patients with advanced non-small cell lung cancer (NSCLC) receiving immune checkpoint inhibitor (ICI)-based therapy.

**Methods:**

We retrospectively enrolled 158 patients with advanced or recurrent NSCLC who received ICI-based therapy at our center. Patients were dichotomized into high- (≥ 120 cells/µL) and low- (< 120 cells/µL) baseline AEC groups. Baseline clinical characteristics, treatment regimens, and immune-related adverse events (irAEs) were collected. Associations between baseline AEC and progression-free survival (PFS) and overall survival (OS) were analyzed using Kaplan–Meier curves and multivariable Cox proportional hazards models. Chi-squared tests were used to compare response rates and irAE incidence between groups. A Fine-Gray competing-risk regression model was applied to identify predictors of ICI-pneumonitis, accounting for death or progression as competing events.

**Results:**

Patients with high baseline AEC showed significantly improved median PFS (mPFS, 24.3 vs. 12.4 months, *p* < 0.001) and median OS (mOS, not reached vs. 48.3 months, *p* = 0.008), together with a higher objective response rate (ORR, 59.1% vs. 40.0%, *p* = 0.018). Multivariable Cox models confirmed high baseline AEC as an independent favorable prognostic factor for both PFS (hazard ratio [HR]: 0.402, 95% confidence interval [CI]: 0.265–0.609; *p* < 0.001) and OS (HR: 0.483, 95% CI: 0.256–0.912; *p* = 0.025). The high-AEC group experienced a higher incidence of any irAEs (39.8% vs. 23.1%, *p* = 0.028) and ICI-pneumonitis (17.2% vs. 4.6%, *p* = 0.017). In the Fine-Gray competing-risk model, high baseline AEC remained independently associated with a higher cumulative incidence of pneumonitis (sHR: 4.33, 95% CI: 1.33–14.02; *p* = 0.015).

**Conclusion:**

Our findings suggest that baseline AEC may serve as a dual biomarker in NSCLC immunotherapy, demonstrating associations with both improved survival outcomes and an elevated risk of ICI-pneumonitis. These results highlight the need for vigilant monitoring in patients with high baseline AEC. Incorporating this readily available parameter into clinical assessment could help refine risk–benefit stratification for patients considering ICI-based therapy.

**Supplementary Information:**

The online version contains supplementary material available at 10.1186/s12885-026-16104-0.

## Introduction

Lung cancer is the leading cause of cancer-related mortality worldwide and the most frequently diagnosed cancer in China. Epidemiological data from 2020 indicate that in China, the incidence rate was 35 per 100,000 people, with a mortality rate of 30.2 per 100,000 [1]. Non-small cell lung cancer (NSCLC) is the most common histological type, accounting for more than 80% of all lung cancer cases. Between 2019 and 2021, the age-standardized five-year relative survival rate for lung cancer remained below 30% [2]. NSCLC places a heavy disease burden on patients, consequently resulting in severe economic and psychological pressure post-diagnosis. The introduction of immunotherapy has dramatically changed this landscape. This shift, marked by the widespread use of immune checkpoint inhibitors (ICIs), has significantly improved the prognosis of NSCLC patients, bringing new hope to their treatment [3–5]. However, the efficacy of immunotherapy exhibits considerable heterogeneity among individuals. In clinical practice, a proportion of NSCLC patients respond poorly and derive limited benefit from this treatment. Moreover, the occurrence of severe immune-related adverse events (irAEs) often complicates therapy and impacts patient prognosis. Hence, the identification of biomarkers capable of predicting both immunotherapy efficacy and irAEs is of paramount importance. It would allow clinicians to better evaluate the potential for patient benefit and facilitate the development of more personalized treatment strategies.

Previous studies have established that patients with high Programmed cell death-ligand 1 (PD-L1) expression and negative driver genes are more likely to benefit from immunotherapy [6]. Nevertheless, treatment efficacy remains inconsistent even within this specific patient subgroup [7]. As such, there is a pressing need to discover novel biomarkers capable of predicting both immunotherapy response and the incidence of irAEs.

Eosinophils are multifunctional leukocytes that contribute to immune defense and regulation, notably in parasitic infections and allergic inflammation. Evolving evidence now underscores their emerging role in cancer. The inflammatory cytokines and chemokines derived from the tumor and its associated immune cells facilitate the recruitment of eosinophils to the tumor microenvironment (TME). Once present, tumor-infiltrating eosinophils can potentially influence cancer progression by directly interacting with tumor cells and by indirectly regulating the anti-tumor immune response, suggesting a dual mechanism of action [8].

The mechanism of lung cancer immunotherapy involves blocking immune checkpoints to counteract tumor-induced immunosuppression, thus restoring anti-tumor immunity. Conversely, irAEs arise from excessive immune activation against healthy tissues. Eosinophils, as key immunomodulatory cells within the tumor microenvironment, are positioned to potentially shape these dual outcomes. This compelling biological link prompted us to hypothesize a role for eosinophils in modulating therapeutic efficacy and risk. To test this, we conducted a retrospective study to evaluate whether pre-treatment absolute eosinophil count (AEC) level can predict treatment response and irAE incidence in NSCLC patients undergoing immunotherapy.

## Materials and methods

### Patients

We retrospectively enrolled patients with advanced or recurrent non-small cell lung cancer (NSCLC) who received PD-1/PD-L1 inhibitor-based therapy at the Department of Respiratory and Critical Care Medicine, the Second Affiliated Hospital of Xi’an Jiaotong University, between January 1, 2022, and December 31, 2023. Inclusion criteria were: ① aged 18 years or older; ② histologically confirmed advanced (stage IIIB-IV) or recurrent non-small cell lung cancer according to the ninth edition of the AJCC TNM staging system; ③ an Eastern Cooperative Oncology Group (ECOG) performance status (PS) score of 0–2; ④ completion of at least four consecutive cycles of PD-1/PD-L1 inhibitor therapy (as monotherapy or in combination with chemotherapy/anti-angiogenic agents); ⑤ presence of at least one radiographically measurable target lesion per Response Evaluation Criteria in Solid Tumors (RECIST) version 1.1; ⑥ availability of complete clinical follow-up data. This study was approved by the Ethics Committee of Xi’an Jiaotong University.

### Data collection

Data on the following clinical characteristics were obtained: demographics (age, gender), clinical profiles (smoking history, ECOG PS, pre-existing lung disease), tumor characteristics (histology, driver mutational status, PD-L1 tumor proportion score [TPS]), treatment details (therapeutic regimen), key systemic inflammatory markers (Neutrophil-to-Lymphocyte Ratio; NLR, Lymphocyte-to-Monocyte Ratio; LMR, and Platelet-to-Lymphocyte Ratio; PLR) and the primary laboratory variable—baseline AEC. Baseline AEC was defined as the AEC measured from the most recent routine blood test prior to the initiation of ICI-based therapy, typically within one week. If multiple tests were available, the result closest to the treatment start date was selected. The final follow-up date was July 15, 2025. The occurrence of irAEs was documented. For patients who experienced irAEs, the type, severity (graded according to the sixth edition of Common Terminology Criteria for Adverse Event, CTCAE), and time of onset were recorded. The best overall response and clinical outcomes for all patients were also collected.

### Statistical analysis

For analytical purposes, patients were stratified into high-AEC (≥ 120 cells/µL) and low-AEC (< 120 cells/µL) groups based on their baseline AEC. The cutoff of 120 cells/µL was selected a priori, as it falls within the range of thresholds used in prior studies of NSCLC immunotherapy [9–11] and corresponds to a clinically established threshold for eosinophilia.

Patient characteristics of two groups were summarized using descriptive statistics respectively. Continuous variables were expressed as medians with interquartile ranges (IQRs), and categorical variables were presented as counts and percentages. *P* values were calculated using the Mann-Whitney U test for continuous variables and Pearson’s chi-squared test or Fisher’s exact test for categorical variables, as appropriate. The primary efficacy endpoints were PFS and OS. PFS was defined as the time from the initiation of immunotherapy to the first documented disease progression or death from any cause, whichever occurred first. OS was defined as the time from the initiation of immunotherapy to death from any cause or last follow-up. Tumor response was evaluated according to RECIST version 1.1. Radiographic re-evaluations were routinely performed at intervals of 6–8 weeks (± 1 week) following treatment initiation until disease progression. The ORR was defined as the proportion of patients achieving a CR or PR. Survival curves were estimated using the Kaplan–Meier method, and differences between groups were compared with the log-rank test.

Associations between clinical variables and survival (PFS/OS) were evaluated using Cox proportional hazards models. The final multivariable models were constructed to adjust for a pre-specified set of clinically and biologically pertinent covariates. These included ECOG PS, pre-existing lung disease, PD-L1 expression level (categorized as < 1%, 1–49%, ≥ 50%, and unknown), baseline systemic inflammatory markers (NLR, LMR, PLR), and baseline AEC (dichotomized at 120 cells/µL). PD-L1 expression was entered into the multivariable Cox models as a single four-category variable (< 1%, 1–49%, ≥ 50%, and unknown), with the < 1% group serving as the reference category. For NLR, LMR, and PLR, we used interquartile range (IQR) scaling to enable clinically meaningful comparisons across variables with different scales. Specifically, each variable was normalized as (value − P25) / IQR, such that the hazard ratio represents the effect of increasing from the 25th to the 75th percentile. AEC was dichotomized using a previously established cutoff of 120 cells/µL based on prior literature, which preserves clinical interpretability. Results are presented as hazard ratios (HRs) with 95% confidence intervals (CIs). Differences in objective response rate (ORR) and the incidence of any irAEs as well as ICI-pneumonitis between the high- and low-AEC groups were compared using Pearson’s chi-squared test or Fisher’s exact test, as appropriate.

Additional pneumonitis modeling was performed to improve robustness under sparse events and to account for competing risks. Because ICI-related pneumonitis was relatively infrequent, Firth penalized logistic regression was used as the primary approach to reduce small-sample bias and mitigate separation; adjusted odds ratios (ORs) with 95% CIs were reported.

To incorporate time-to-event information and the fact that progression or death may preclude the observation of pneumonitis, a Fine-Gray competing-risk regression model was fitted with pneumonitis as the event of interest and progression/death as competing events; subdistribution hazard ratios (sHRs) with 95% CIs were reported.

A 56-day landmark sensitivity analysis was pre-specified by restricting to patients without pneumonitis or competing events before day 56 and re-originating time at day 56.

Data management and statistical analyses were performed using R (≥ 4.2), SPSS (version 30.0, IBM Corp.), and GraphPad Prism (version 10.5, GraphPad Software). A two-sided *p* value < 0.05 was considered statistically significant.

## Results

### Patient characteristics

We retrospectively analyzed a cohort of 158 patients with advanced or recurrent NSCLC who received ICI-based treatment within the specified study period and met all inclusion criteria. The demographic and clinical characteristics of the study population are summarized in Table 1. Baseline characteristics were compared between patients with high- and low- AEC group, and the two groups were generally well balanced. There were no statistically significant differences in age, gender, smoking history, ECOG performance status, tumor histology, PD-L1 expression categories, or the line of ICI-based therapy between the two groups (all *p* > 0.05). Critically, key systemic inflammatory markers—including NLR, LMR, and PLR—were also comparable at baseline (all *p* > 0.05). The prevalence of pre-existing lung disease and the distribution of oncogenic driver mutations were similarly balanced. As per the stratification design, baseline AEC was markedly lower in the low-AEC group than in the high-AEC group (median, 50.0 [IQR 20.0–70.0] vs. 200.0 [IQR 150.0–310.0] cells/µL, *p* < 0.001).

**Table 1:** Comparison of baseline characteristics, comorbidities, immune-related adverse events, and driver mutations between high- and low-AEC groups

Variable	Category	High-AEC (≥ 120 cells/µL)	Low-AEC (< 120 cells/µL)	*p* value
Patients, n		93	65	
Age, years		63.0 (58.0–68.0)	62.0 (57.0–68.0)	0.696
Gender	Female	9 (9.7%)	7 (10.8%)	1.000
	Male	84 (90.3%)	58 (89.2%)	
Smoking history	Yes	56 (60.2%)	34 (52.3%)	0.410
	No	37 (39.8%)	31 (47.7%)	
Histology	Adenocarcinoma	36 (38.7%)	25 (38.5%)	0.332
	Squamous	52 (55.9%)	39 (60.0%)	
	Other	5 (5.3%)	1 (1.5%)	
ECOG PS	0	77 (82.8%)	59 (90.8%)	0.381
	1	14 (15.1%)	5 (7.7%)	
	≥ 2	2 (2.2%)	1 (1.5%)	
Therapy line at ICI	1st line	79 (84.9%)	58 (89.2%)	0.298
	≥ 2nd line	14 (15.1%)	7 (10.8%)	
Pre-existing lung disease	Yes	26 (28.0%)	11 (16.9%)	0.155
	No	67 (72.0%)	54 (83.1%)	
PD-L1 expression	< 1%	17 (18.3%)	13 (20.0%)	0.337
	1–49%	23 (24.7%)	21 (32.3%)	
	≥ 50%	19 (20.4%)	12 (18.5%)	
	Unknown	34 (36.6%)	19 (29.2%)	
Driver mutation	Positive	10 (10.8%)	4 (6.1%)	0.616
	Negative	24 (25.8%)	14 (21.5%)	
	Unknown	59 (63.4%)	47 (72.3%)	
Baseline NLR		3.43 (2.05–4.98)	3.22 (2.30–5.08)	0.899
Baseline LMR		2.72 (2.00-4.15)	3.14 (1.92–4.52)	0.601
Baseline PLR		142.06 (107.37-206.94)	164.87 (117.88-238.67)	0.117
Baseline AEC, cells/µL		200.0 (150.0–310.0)	50.0 (20.0–70.0)	< 0.001

Data are presented as n (%) or median (interquartile range), as appropriate. *P* values were calculated using the Mann-Whitney U test for continuous variables and Pearson’s chi-squared test or Fisher’s exact test for categorical variables, as appropriate. High-AEC: baseline AEC ≥ 120 cells/µL; low-AEC: baseline AEC < 120 cells/µL

*Abbreviations: AEC* absolute eosinophil count,* ECOG PS* Eastern Cooperative Oncology Group performance status, *PD-L1* programmed death-ligand 1, *NLR* Neutrophil-to-Lymphocyte Ratio, *LMR* Lymphocyte-to-Monocyte Ratio, *PLR* Platelet-to-Lymphocyte Ratio

### Correlation between baseline AEC and clinical outcomes

**Fig. 1:**
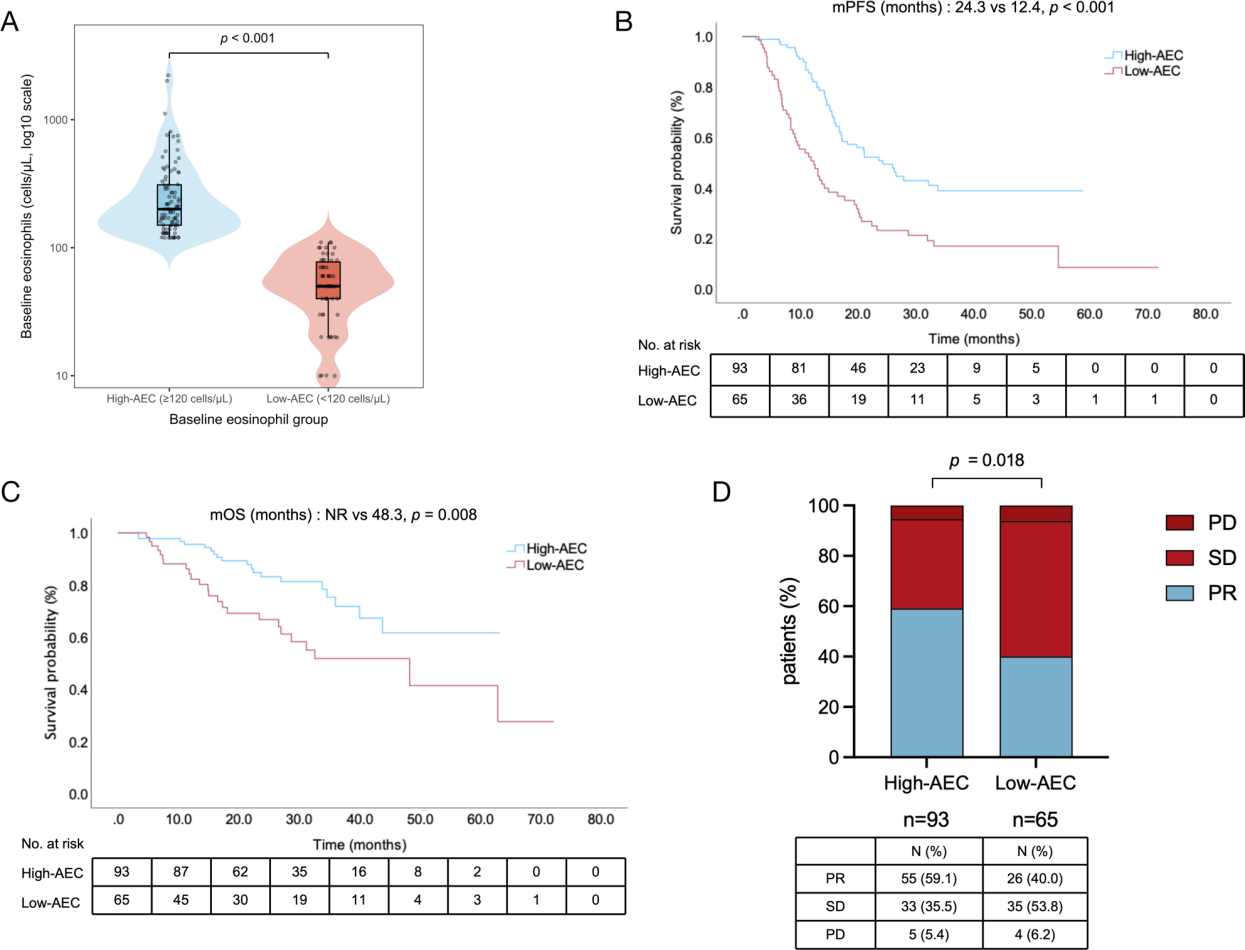
Baseline AEC and the efficacy of ICI-based therapy in NSCLC patients (*n* = 158). **A** Distribution of baseline AEC in the overall cohort, displayed on a log10 scale. **B** Kaplan–Meier curves for PFS according to baseline AEC group (high vs. low). The number of patients at risk over time is shown below the x-axis. **C** Kaplan–Meier curves for OS stratified by baseline AEC group, with the corresponding number at risk displayed beneath the plot. **D** Distribution of best overall response according to baseline AEC group, illustrated as a stacked bar chart including PR, SD, and PD. P values are derived from log-rank tests for PFS and OS and from chi-squared tests for response rates. Abbreviations: AEC, absolute eosinophil count; ICI, immune checkpoint inhibitor; NSCLC, non-small cell lung cancer; PFS, progression-free survival; OS, overall survival; CR, complete response; PR, partial response; SD, stable disease; PD, progressive disease; NR, not reached

The distribution of baseline AEC in the overall cohort is shown in Fig. 1A (median 130 cells/µL, IQR 60.0–217.5). Survival analyses revealed a significant benefit in favor of the high-AEC group. Specifically, the median PFS (mPFS) was 24.3 months in the high-AEC group compared with 12.4 months in the low-AEC group (*p* < 0.001, Fig. 1B). The OS analysis showed an even more pronounced advantage: the median OS (mOS) was not reached in the high-AEC group, whereas it was 48.3 months in the low-AEC group (*p* = 0.008, Fig. 1C). Consistent with these findings, the ORR was significantly higher in the high-AEC group than in the low-AEC group (59.1% vs. 40.0%, *p* = 0.018, Fig. 1D), further underscoring the association between elevated baseline eosinophils and improved treatment outcomes.

### Univariate and multivariate analyses of prognostic factors for PFS and OS

For PFS, univariate Cox regression identified high-baseline AEC as a significant predictor for favorable outcome (HR: 0.432, 95% CI: 0.292–0.638; *p* < 0.001, Table 2). This association remained robust in the multivariate analysis after adjusting for potential confounders (adjusted HR: 0.402, 95% CI: 0.265–0.609; *p* < 0.001). In contrast, other clinical variables—including age, gender, smoking history, histology type, line of treatment, and baseline inflammatory markers (NLR, LMR, PLR) were not significantly associated with PFS in univariate analyses. Variables demonstrating a potential association with PFS in univariate analysis (*p* ≤ 0.20), along with clinically relevant factors (PD-L1 expression) and key biomarkers of interest (baseline inflammatory indices), were incorporated into a multivariable Cox proportional hazards model. In this comprehensive model, a high baseline AEC (HR: 0.402, 95% CI: 0.265–0.609; *p* < 0.001) and PD-L1 expression ≥ 50% (HR: 0.558, 95% CI: 0.333–0.938; *p* = 0.028) were identified as independent factors associated with prolonged PFS, whereas an ECOG PS ≥ 1 was associated with an increased risk of progression (HR: 1.827, 95% CI: 1.016–3.284; *p* = 0.044). Baseline NLR, LMR, PLR, and pre-existing lung disease did not retain independent prognostic significance in the multivariable analysis.

**Table 2:** Univariate and Multivariate Cox regression analysis for PFS in all patients (n=158)

Variables	Univariate Analysis			Multivariate Analysis		
	HR	95% CI	*p* value	HR	95% CI	*p* value
Age (≥65 vs. <65)	0.881	0.594-1.307	0.529			
Gender (male vs. female)	0.692	0.386-1.240	0.216			
Smoking history (yes vs. no)	0.792	0.536-1.170	0.242			
Histology type (adeno vs. others)	1.017	0.685-1.511	0.933			
EOCG PS (≥1 vs.0)	1.483	0.843-2.609	0.172	1.827	1.016-3.284	0.044
Line of treatment (≥2nd line vs. 1st line)	1.256	0.711-2.219	0.433			
Pre-existing lung disease (yes vs. no)	0.606	0.385-0.953	0.03	0.709	0.442-1.136	0.152
PD-L1 expression (1-49% vs. 1%)	0.956	0.559-1.633	0.868	1.092	0.631-1.889	0.753
PD-L1 expression (≥50% vs. 1%)	0.634	0.383-1.049	0.076	0.558	0.333-0.938	0.028
PD-L1 expression (Unknown vs. 1%)	0.636	0.361-1.120	0.117	0.663	0.372-1.181	0.163
Baseline NLR (per IQR)	0.960	0.793-1.161	0.672	0.975	0.777-1.222	0.825
Baseline LMR (per IQR)	0.938	0.809-1.089	0.403	0.981	0.832-1.158	0.823
Baseline PLR (per IQR)	0.965	0.782-1.190	0.740	0.956	0.729-1.255	0.747
Baseline AEC (high vs. low)	0.432	0.292-0.638	<0.001	0.402	0.265-0.609	<0.001

For OS, univariate analysis identified high baseline AEC as a significant favorable prognostic factor (HR: 0.448, 95% CI: 0.244–0.822; *p* = 0.009) Table 3. In the subsequent multivariable Cox model, which adjusted for ECOG PS, pre-existing lung disease, PD-L1 expression, and baseline inflammatory markers (NLR, LMR, PLR), high baseline AEC remained an independent predictor of improved OS (adjusted HR: 0.483, 95% CI: 0.256–0.912; *p* = 0.025). Additionally, an ECOG PS ≥ 1 was independently associated with worse OS (adjusted HR: 2.354, 95% CI: 1.037–5.342; *p* = 0.041). No other variables, including PD-L1 expression or baseline inflammatory indices, demonstrated independent prognostic significance for OS in the multivariate analysis.

**Table 3:** Univariate and Multivariate Cox regression analysis for OS in all patients (*n* = 158)

Variables	Univariate Analysis			Multivariate Analysis			
	HR	95% CI	*p* value	HR	95% CI	*p* value	
Age (≥ 65 vs. <65)	1.15	0.629–2.120	0.65				
Gender (male vs. female)	0.67	0.282–1.592	0.365				
Smoking history (yes vs. no)	0.836	0.457–1.528	0.56				
Histology type (adeno vs. others)	0.728	0.387–1.369	0.324				
ECOG PS (≥ 1 vs.0)	2.125	0.983–4.597	0.055	2.354	1.037–5.342		0.041
Line of treatment (≥ 2nd line vs. 1st line)	1.125	0.474–2.670	0.79				
Pre-existing lung disease (yes vs. no)	0.544	0.268–1.106	0.093	0.571	0.274–1.191		0.135
PD-L1 expression (1–49% vs. 1%)	0.634	0.230–1.750	0.379	0.699	0.253–1.936		0.491
PD-L1 expression (≥ 50% vs. 1%)	1.127	0.551–2.305	0.744	1.208	0.574–2.541		0.618
PD-L1 expression (Unknown vs. 1%)	0.777	0.328–1.804	0.566	0.899	0.365–2.213		0.816
Baseline NLR (per IQR)	0.784	0.543–1.131	0.193	0.698	0.426–1.144		0.153
Baseline LMR (per IQR)	0.909	0.702–1.177	0.470	0.822	0.571–1.183		0.292
Baseline PLR (per IQR)	0.977	0.710–1.345	0.889	1.184	0.744–1.884		0.475
Baseline AEC (high vs. low)	0.448	0.244–0.822	0.009	0.483	0.256–0.912		0.025

Variables with a *p* value ≤ 0.2 in the univariate analysis were included in the multivariate Cox proportional hazards models. In addition, PD-L1 expression level and key systemic inflammatory markers (including NLR, LMR and PLR) were forced into the final multivariate models regardless of their univariate *p* values to specifically address their potential confounding effect on the association between baseline AEC and survival outcomes

*Abbreviations: PFS* progression-free survival, *HR* hazard ratio, *CI* confidence interval, *adeno* adenocarcinoma, *ECOG PS* Eastern Cooperative Oncology Group performance status, *PD-L1* programmed death-ligand 1, *NLR* Neutrophil-to-Lymphocyte Ratio, *LMR* Lymphocyte-to-Monocyte Ratio, *PLR* Platelet-to-Lymphocyte Ratio, *AEC* absolute eosinophil count, *OS* overall survival

### Sensitivity analysis for the AEC cutoff value

To evaluate the robustness of the primary AEC stratification (cutoff: 120 cells/µL), we performed sensitivity analyses using alternative thresholds of 100 and 150 cells/µL. Kaplan-Meier analyses confirmed consistent and significant survival benefits for the high-AEC group in both PFS and OS across the 100 and 120 cells/µL cutoffs (all log-rank *p* < 0.05; see Supplementary Table S1). In multivariable Cox models, the association between high baseline AEC and improved progression-free survival (PFS) remained statistically significant across all tested cutoffs. Regarding overall survival (OS), the favorable association held at the 100 and 120 cells/µL cutoffs in these models, but was attenuated and lost statistical significance at 150 cells/µL (see Supplementary Table S2). This observation suggests that the 120 cells/µL cutoff effectively identifies a patient subset with a distinct immunobiological profile and survival benefit, whereas a higher threshold may dilute this signal.

### Correlation between baseline AEC and the risk of irAEs

**Fig. 2:**
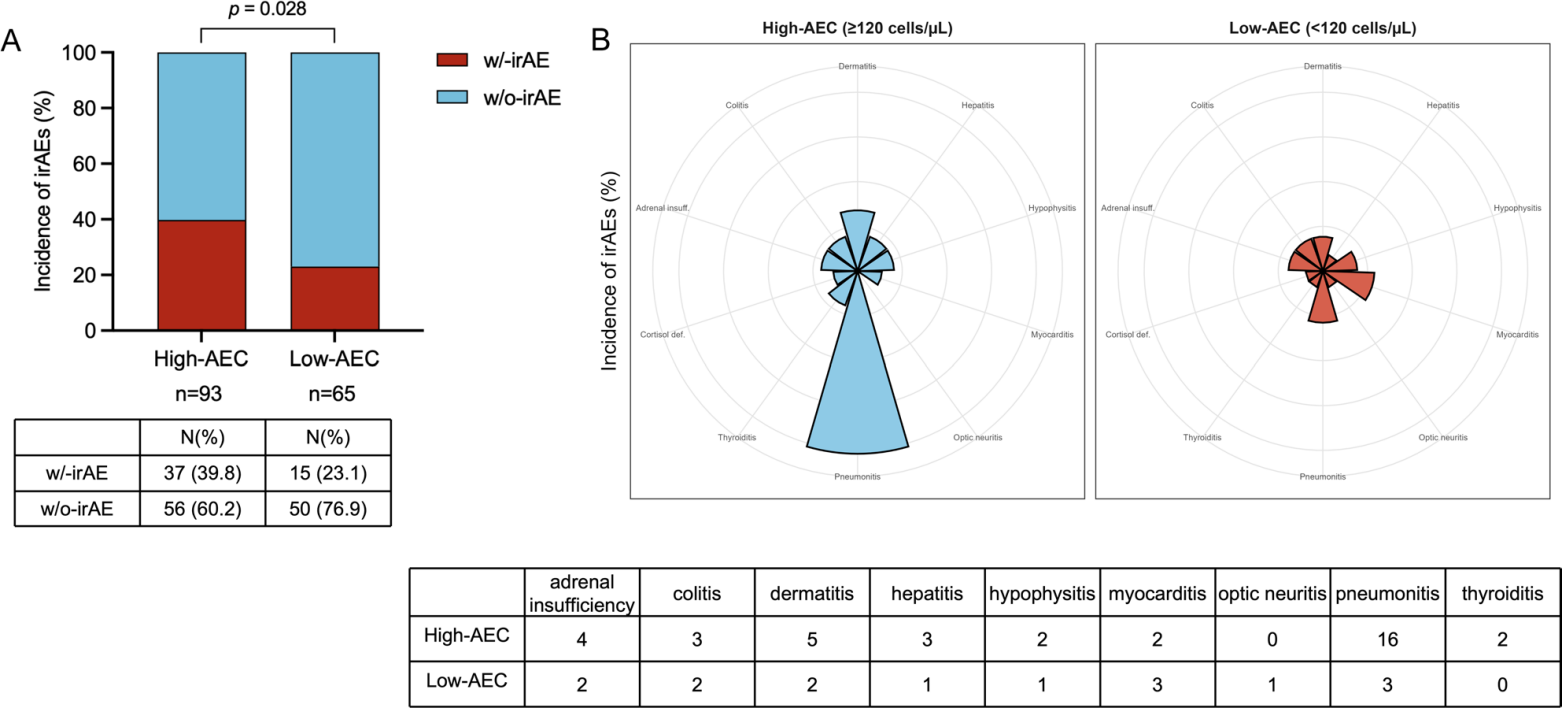
Baseline AEC as a predictor of irAEs in NSCLC patients (*n* = 158). **A** Incidence of any irAEs in patients with high vs. low baseline AEC. Bars represent the proportions of patients with or without irAEs in each AEC group (high vs. low), together with the corresponding *p* value from a chi-squared test. **B** Distribution of irAE subtypes according to baseline AEC group. The rose (radar) plot depicts the relative frequencies of individual irAE categories—including ICI-pneumonitis, dermatitis, colitis, hepatitis, adrenal insufficiency, hypophysitis, thyroiditis, myocarditis, and optic neuritis—in the low- and high-AEC groups. Abbreviations: AEC, absolute eosinophil count; irAEs, immune-related adverse events; NSCLC, non-small cell lung cancer; ICI-pneumonitis, immune checkpoint inhibitor-related pneumonitis; w/ irAE, with irAE; w/o irAE, without irAE

**Fig. 3:**
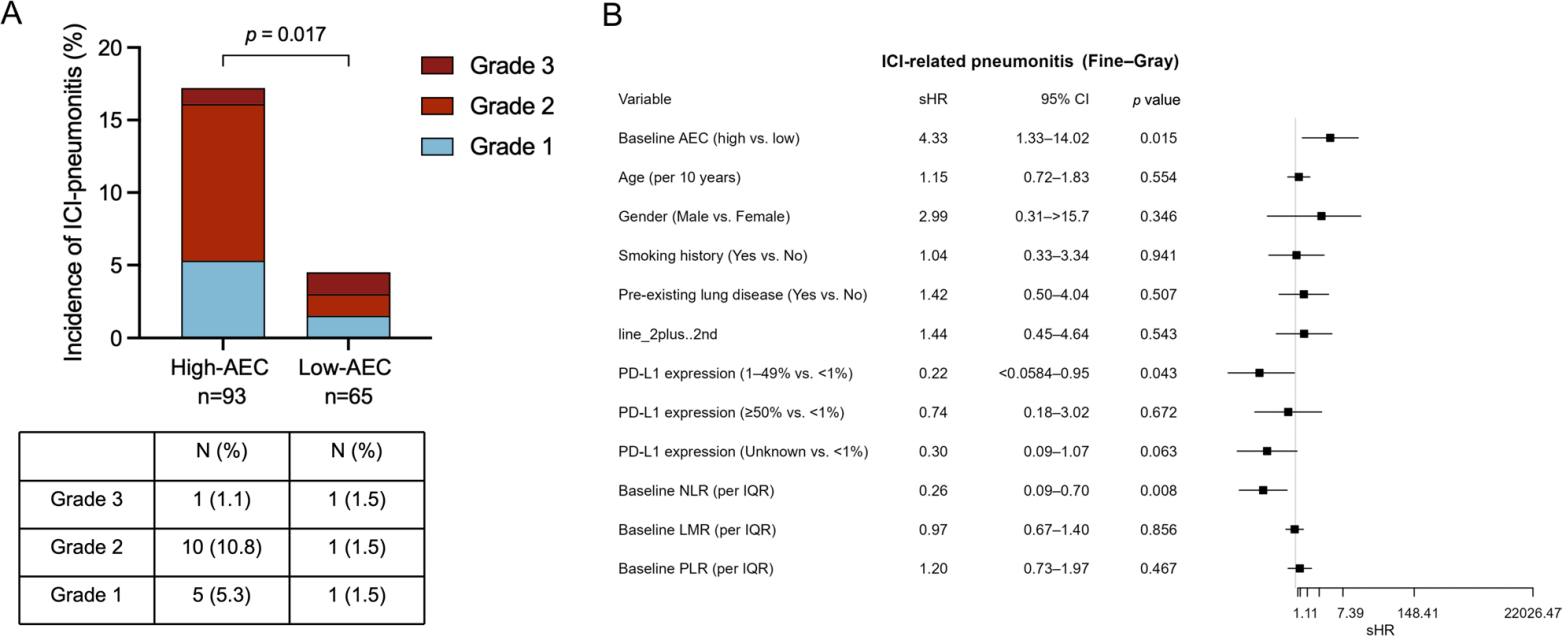
Predictors of ICI-pneumonitis: incidence and competing-risk analysis. **A** Incidence of ICI-pneumonitis stratified by baseline AEC. Stacked bar chart illustrating the proportion of patients who developed ICI-pneumonitis, stratified by baseline AEC group (high vs. low). Each bar is segmented to represent the distribution according to CTCAE grade. The *p* value was derived from a chi-squared test comparing the overall incidence between groups. **B** Forest plot of factors associated with ICI-related pneumonitis using Fine-Gray competing-risk model. sHRs with 95% CI are shown. High baseline AEC remained significantly associated with increased pneumonitis risk after accounting for death or progression as competing events. Abbreviations: ICI-pneumonitis, immune checkpoint inhibitor-related pneumonitis; AEC, absolute eosinophil count; CTCAE, Common Terminology Criteria for Adverse Events; sHRs, Subdistribution hazard ratios; CI, confidence interval

The incidence of any irAEs was numerically higher in the high-AEC group compared with the low-AEC group (39.8% vs. 23.1%, *p* = 0.028, Fig. 2A). With respect to irAE subtypes, immune-related pneumonitis was notably more frequent in the high-AEC group (16 cases, 17.2%) than in the low-AEC group (3 cases, 4.6%; *p* = 0.017, detailed in Fig. 3A). Other irAE subtypes also showed numerically higher counts in the high-AEC group, including dermatitis (5 vs. 2 cases), immune-related colitis (3 vs. 2 cases), hepatitis (3 vs. 1 case), adrenal insufficiency (4 vs. 2 cases), hypophysitis (2 vs. 1 cases), and thyroiditis (2 vs. 0 case). In contrast, myocarditis occurred in 2 patients in the high-AEC group and 3 patients in the low-AEC group, while optic neuritis was observed only in 1 low-AEC patient. The detailed distribution of irAE subtypes according to baseline AEC group is depicted in Fig. 2B.

### Fine-gray competing-risk regression for ICI-pneumonitis

In a competing-risk framework (Fine-Gray model) with progression/death treated as competing events, high baseline AEC was consistently associated with a higher cumulative incidence of pneumonitis (sHR 4.33, 95% CI 1.33–14.02; *p* = 0.015; Fig. 3B). Baseline NLR was inversely associated with pneumonitis (sHR 0.26, 95% CI 0.09–0.70; *p* = 0.008), and PD-L1 1–49% (vs. <1%) was associated with a lower pneumonitis incidence (sHR 0.22, 95% CI 0.05–0.95; *p* = 0.043) (Supplementary Table S4).

As a sensitivity analysis to address sparse-event bias, we additionally applied Firth penalized multivariable logistic regression. High baseline AEC remained independently associated with increased pneumonitis risk (OR 3.80, 95% CI 1.23–15.33; *p* = 0.018; Supplementary Figure S4). Baseline NLR was again inversely associated (OR 0.35, 95% CI 0.10–0.91; *p* = 0.028), while other covariates showed no significant association (Supplementary Table S4).

In the 56-day landmark sensitivity analysis, no pneumonitis events occurred after day 56 among patients event-free at the landmark; thus, the landmark model was not estimable. This finding suggests that pneumonitis events clustered early after ICI initiation in this cohort.

## Discussion

Eosinophils are derived from CD34 + pluripotent progenitor cells in the bone marrow and normally account for only 1%–6% of circulating leukocytes [12]. Their role in asthma and COPD has been extensively investigated, and eosinophil-targeting biologics have become an integral part of the management of type 2 airway inflammation [13, 14]. In contrast, their function in cancer—particularly in the setting of immunotherapy—is less clearly defined, although accumulating data suggest that eosinophils exert diverse and context-dependent effects within the TME [15]. Against this background, we investigated whether a simple, routinely available parameter—baseline AEC—could serve as a clinically useful biomarker to predict both efficacy and toxicity of ICI-based therapy in NSCLC.

In our cohort of 158 advanced or recurrent NSCLC patients treated with ICIs, a high baseline AEC (≥ 120 cells/µL) was independently associated with improved PFS and OS in multivariable analyses. Consistently, patients in the high-AEC group also achieved a significantly higher ORR. These findings are consistent with the tumor-suppressive arm of the eosinophil literature. Experimental work has shown that eosinophils can directly kill tumor cells through contact-dependent degranulation of cytotoxic molecules such as eosinophil cationic protein, eosinophil peroxidase, and granzyme B after activation by alarmins such as IL-33 [12, 15, 16]. IL-33-activated eosinophils adhere to tumor cells, polarize effector proteins and CD11b/CD18 to the immune synapse, and induce tumor cell death [16, 17]. Eosinophils can also restrict metastasis and reshape the TME by recruiting and supporting CD4⁺ and CD8⁺ T-cell infiltration, as well as by promoting vascular normalization and suppressing angiogenesis [18–21]. Within this mechanistic framework, our clinical observation that higher peripheral AEC predicts better ICI outcomes in NSCLC supports the hypothesis that eosinophils contribute to a more “immunologically permissive” TME, thereby amplifying the anti-tumor effects of PD-1/PD-L1 blockade.

At the same time, our data highlight the potential “cost” of this favorable immune milieu. We observed a numerically higher overall incidence of irAEs in the high-AEC group (39.8% vs. 23.1%), and a significantly increased risk of ICI-pneumonitis (17.2% vs. 4.5%). Both Firth penalized logistic regression and Fine-Gray competing-risk analysis confirmed baseline AEC as an independent predictor of ICI-pneumonitis, with consistent effect sizes across models (OR 3.80, sHR 4.33). Baseline NLR was inversely associated with pneumonitis risk in both models, while PD-L1 1–49% showed a protective effect only in the Fine-Gray framework. These findings align with experimental evidence that eosinophils can promote organ-specific inflammation under certain conditions. Distinct eosinophil programs have been shown either to suppress or to facilitate metastasis: IL-5-regulated eosinophils can inhibit pulmonary metastasis, whereas alternative IL-5–driven eosinophil responses can promote lung metastasis and metastatic niche formation, partly through chemokines such as CCL6 and interactions with other myeloid cells [18, 22, 23]. Eosinophils can also support tumor growth and angiogenesis via cytokines and growth factors, as reported in astrocytoma and cervical cancer models [24–26]. More recently, eosinophil-derived IL-4 has been implicated in driving immunosuppressive myeloid cell differentiation in the TME; genetic or pharmacologic targeting of the IL-4/IL-4 receptor axis (e.g., with dupilumab) can reduce myeloid suppression and enhance CD8⁺ T-cell–mediated tumor control, synergizing with ICIs [27]. Taken together, these mechanistic studies suggest that eosinophils occupy a central position at the interface between anti-tumor immunity and immune-mediated tissue injury. Our clinical data extend this concept to NSCLC immunotherapy by showing that a higher baseline AEC marks both stronger anti-tumor efficacy and greater susceptibility to lung-specific immune toxicity.

To address the methodological challenges of rare events and competing risks in pneumonitis analysis, we employed Firth penalized logistic regression and Fine-Gray competing-risk models. The consistent association of baseline AEC with pneumonitis across both approaches strengthens confidence in the robustness of our findings. Additionally, pneumonitis events occurred predominantly within the first 56 days of ICI therapy, suggesting that intensified monitoring during this early period may be particularly important for patients with high baseline AEC.

Notably, the predictive value of PD-L1 varied across endpoints: PD-L1 ≥ 50% independently predicted PFS but not OS, while PD-L1 1–49% showed a protective effect against pneumonitis in the Fine-Gray model. This dissociation suggests that determinants of initial response, long-term survival, and toxicity may differ. Interestingly, lower baseline NLR was consistently associated with higher pneumonitis risk in both models but had no prognostic value for survival, further supporting that distinct biological pathways underlie efficacy and toxicity.

The strengths and novelty of our study lie in several key aspects. First, we validated the dual role of baseline AEC within a comprehensive multivariable framework. Critically, the prognostic value of high baseline AEC for both PFS and OS remained significant after rigorous adjustment for PD-L1 expression and systemic inflammatory indices (NLR, LMR, PLR). Of note, while PD-L1 ≥ 50% was independently associated with improved PFS, none of the inflammatory markers showed significant independent association with either survival endpoint. This indicates that AEC captures a distinct immunobiology beyond general inflammation or PD-L1 status.

Sensitivity analyses using alternative cutoffs (100 and 150 cells/µL) supported the choice of 120 cells/µL. While the association between high AEC and improved PFS was robust across all thresholds, the OS benefit was maintained at 100 and 120 cells/µL but attenuated at 150 cells/µL, suggesting that 120 cells/µL optimally identifies patients with a clinically meaningful eosinophil-associated survival advantage.

Our analysis also provides nuanced insights into traditional prognostic factors. While PD-L1 expression ≥ 50% was an independent favorable factor for PFS, it did not predict OS. This may reflect the complexity of long-term survival determinants, including the influence of subsequent therapies, as well as the potential dilution of PD-L1’s predictive power by concurrent chemotherapy in many first-line combination regimens. Conversely, ECOG PS consistently emerged as a strong independent adverse factor for both endpoints, reaffirming its fundamental role in patient outcomes.

These findings have direct clinical implications. Baseline AEC is derived from a routine complete blood count—inexpensive, ubiquitous, and easily integrated into clinical workflows. In practice, baseline AEC could contribute to personalized risk–benefit assessment: patients with high AEC may be prioritized for ICI-based therapy given their higher likelihood of durable benefit, yet should also be flagged for enhanced pulmonary monitoring due to their elevated pneumonitis risk.

The link between high AEC and increased pneumonitis risk may reflect eosinophils’ role in type-2 immunity and lung-specific vulnerability in NSCLC, particularly in patients with prior smoking or subclinical interstitial changes. For such patients, we recommend heightened vigilance—including closer symptom monitoring, a lower threshold for diagnostic chest CT, and earlier corticosteroid initiation if pneumonitis is suspected—to preserve efficacy while mitigating severe toxicity.

Several limitations should be acknowledged. First, the single-center, retrospective design with a moderate sample size limits causal inference and may introduce selection bias. Second, only a single baseline AEC measurement was available; longitudinal dynamics during treatment were not assessed, which could provide valuable insights into the temporal relationship between eosinophil kinetics and clinical outcomes. Third, although we selected the 120 cells/µL cutoff based on prior literature and supported it with sensitivity analyses, validation in larger, prospective cohorts across diverse populations is needed. Fourth, driver mutation status was unknown in 63.8% of patients, precluding a robust sensitivity analysis to confirm the independence of AEC from driver mutations. Fifth, due to the limited number of pneumonitis events, detailed grade-specific analyses were not feasible. Future studies with systematic collection of clinical outcomes, such as corticosteroid use and long-term resolution, are warranted. Finally, the observational nature of this study precludes definitive conclusions about the causal biological pathways linking baseline eosinophils to both enhanced efficacy and organ-specific toxicity.

## Conclusion

This study supports the potential of baseline AEC as a practical, readily available dual-signal biomarker in NSCLC patients receiving ICIs, simultaneously indicating a higher likelihood of durable survival benefit and an elevated risk of immune-related pneumonitis. Notably, the association with survival outcomes remained significant after adjustment for key clinical factors and systemic inflammatory indices, underscoring its independent value. Future prospective, multi-center studies are warranted to validate these findings, to define optimal cutoffs and the significance of on-treatment dynamics, and to elucidate the underlying biology linking eosinophils to anti-tumor efficacy and lung-specific toxicity. Incorporating this accessible parameter into clinical decision-making could aid in personalized risk–benefit assessments, potentially guiding more tailored monitoring and management strategies for NSCLC patients undergoing immunotherapy.

## Supplementary Information

Supplementary Material 1.

## Data Availability

The de-identified individual participant data that underlie the results reported in this article will be made available upon reasonable request to the corresponding author.

## Abbreviations

NSCLC: Non-small cell lung cancer
ICIs: Immune checkpoint inhibitors
irAEs: Immune-related adverse events
PD-L1: Programmed cell death-ligand 1
TME: Tumor microenvironment
AEC: Absolute eosinophil count
ECOG PS: Eastern Cooperative Oncology Group performance status
PR: Partial response
SD: Stable disease
PD: Progressive disease
RECIST: Response Evaluation Criteria in Solid Tumors
ORR: Objective response rate
CR: Complete response
TPS: Tumor proportion score
CTCAE: Common Terminology Criteria for Adverse Event
IQRs: Interquartile ranges
PFS: Progression-free survival
OS: Overall survival
HR: Hazard ratio
CI: Confidence interval
OR: Odds ratio
COPD: Chronic obstructive pulmonary disease
