# Supplementary material for: The dual role of baseline absolute eosinophil count in non-small cell lung cancer immunotherapy: a biomarker for enhanced efficacy and elevated risk of immune checkpoint inhibitor-related pneumonitis
Source: BMC Cancer. 2026 Apr 27;26:738. doi: 10.1186/s12885-026-16104-0 (PMC13251072; doi:10.1186/s12885-026-16104-0)
Supplement: Supplementary file 1 — Supplementary Material 1. [file 12885_2026_16104_MOESM1_ESM.docx]

**Supplementary Table S1.** Survival outcomes stratified by alternative baseline AEC cut-off values.

| **AEC cutoff (cells/μL)** | **Group** | **mPFS (months)** | **log-rank p value** | **mOS (months)** | **log-rank p value** |
| --- | --- | --- | --- | --- | --- |
| 100 | High-AEC | 24.3 | <0.001 | NR | 0.001 |
|  | Low-AEC | 11.5 |  | 32.6 |  |
| 120 (primary) | High-AEC | 24.3 | <0.001 | NR | 0.008 |
|  | Low-AEC | 12.4 |  | 48.3 |  |
| 150 | High-AEC | 26 | <0.001 | NR | 0.034 |
|  | Low-AEC | 13.8 |  | 48.3 |  |

Notes: Survival comparisons were performed using the Kaplan–Meier method with log-rank tests. The primary analysis cut-off was 120 cells/μL. Sensitivity analyses using cut-offs of 100 and 150 cells/μL are presented to demonstrate the robustness of the association between high baseline AEC and improved survival outcomes.

Abbreviations: AEC, absolute eosinophil count; mPFS, median progression-free survival; mOS, median overall survival; NR, not reached.

**Supplementary Table S2.** Multivariable Cox regression analyses of PFS and OS using different cut-offs for baseline AEC.

| **AEC cutoff (cells/μL)** | **End point** | **HR** | **95% CI** | ***p* value** |
| --- | --- | --- | --- | --- |
| 100 | PFS | 0.351 | 0.232-0.530 | <0.001 |
|  | OS | 0.383 | 0.202-0.726 | 0.003 |
| 120 (primary) | PFS | 0.402 | 0.265-0.609 | <0.001 |
|  | OS | 0.483 | 0.256-0.912 | 0.025 |
| 150 | PFS | 0.501 | 0.324-0.774 | 0.002 |
|  | OS | 0.640 | 0.327-1.251 | 0.192 |

Notes: Multivariable Cox proportional hazards models were adjusted for ECOG PS, pre-existing lung disease, PD-L1 expression level and baseline inflammatory markers (neutrophil-to-lymphocyte ratio, lymphocyte-to-monocyte ratio, and platelet-to-lymphocyte ratio). The primary analysis cut-off was 120 cells/μL. The results demonstrate that a high baseline AEC remains an independent favorable prognostic factor for PFS across all tested cut-offs (100, 120, and 150 cells/μL).

Abbreviations: AEC, absolute eosinophil count; HR, hazard ratio; CI, confidence interval; PFS, progression-free survival; OS, overall survival; ECOG PS, Eastern Cooperative Oncology Group performance status; NLR, Neutrophil-to-lymphocyte ratio; LMR, lymphocyte-to-monocyte ratio; PLR, platelet-to-lymphocyte ratio.

**Supplementary Table S3.** Multivariable Cox regression analyses in the subgroup of patients with known PD-L1 expression status.

| Variables | Multivariate Analysis (PFS) | | | Multivariate Analysis (OS) | | |
| --- | --- | --- | --- | --- | --- | --- |
|  | HR | 95% CI | *p* value | HR | 95% CI | *p* value |
| ECOG PS (≥1 vs.0) | 2.239 | 1.054-4.755 | 0.036 | 2.108 | 0.721-6.167 | 0.173 |
| Pre-existing lung disease (yes vs. no) | 0.637 | 0.362-1.120 | 0.117 | 0.568 | 0.237-1.359 | 0.204 |
| PD-L1 expression (1-49% vs. 1%) | 1.442 | 0.709-2.933 | 0.312 | 0.792 | 0.239-2.622 | 0.703 |
| PD-L1 expression (≥50% vs. 1%) | 0.828 | 0.427-1.607 | 0.578 | 1.176 | 0.466-2.970 | 0.731 |
| Baseline NLR (per IQR) | 1.111 | 0.857-1.439 | 0.426 | 0.789 | 0.411-1.512 | 0.474 |
| Baseline LMR (per IQR) | 1.062 | 0.885-1.274 | 0.517 | 0.950 | 0.662-1.363 | 0.780 |
| Baseline PLR (per IQR) | 0.844 | 0.610-1.168 | 0.306 | 0.984 | 0.538-1.802 | 0.959 |
| Baseline AEC (high vs. low) | 0.172 | 0.026-1.159 | 0.071 | 0.272 | 0.117-0.632 | 0.002 |

Notes:This sensitivity analysis was restricted to the 105 patients with known PD-L1 expression status. Multivariable Cox proportional hazards models for both PFS and OS were constructed using this subgroup and adjusted for all variables listed. Notably, baseline AEC remained a significant independent favorable prognostic factor for OS in this subgroup, further underscoring the robustness of its association with long-term survival benefit from immunotherapy.

Abbreviations: CI, confidence interval; HR, hazard ratio; ECOG PS, Eastern Cooperative Oncology Group performance status; NLR, neutrophil-to-lymphocyte ratio; LMR, lymphocyte-to-monocyte ratio; PLR, platelet-to-lymphocyte ratio; AEC, absolute eosinophil count; PFS, progression-free survival; OS, overall survival.

**Supplementary Table S4.** Multivariable models for ICI-related pneumonitis: Firth penalized logistic regression and Fine–Gray competing-risk regression

| **Variable** | **Firth OR** | **95% CI (OR)** | ***p* value (OR)** | **Fine–Gray sHR** | **95% CI (sHR)** | ***p* value (sHR)** |
| --- | --- | --- | --- | --- | --- | --- |
| Baseline AEC (high vs. low) | 3.80 | 1.23–15.33 | 0.018 | 4.33 | 1.33–14.02 | 0.015 |
| Age (per 10 years) | 1.16 | 0.64–2.15 | 0.623 | 1.15 | 0.72–1.83 | 0.554 |
| Gender (Male vs. Female) | 1.84 | 0.31–20.68 | 0.530 | 2.99 | 0.31–29.18 | 0.346 |
| Smoking history (Yes vs. No) | 1.20 | 0.43–3.63 | 0.734 | 1.04 | 0.33–3.34 | 0.941 |
| Pre-existing lung disease (Yes vs. No) | 1.63 | 0.56–4.56 | 0.359 | 1.42 | 0.50–4.04 | 0.507 |
| Line of treatment (≥2nd line vs. 1st) | 1.35 | 0.30–5.03 | 0.678 | 1.44 | 0.45–4.64 | 0.543 |
| PD-L1 expression (1–49% vs. <1%) | 0.30 | 0.07–1.17 | 0.083 | 0.22 | 0.05–0.95 | 0.043 |
| PD-L1 expression (≥50% vs. <1%) | 0.70 | 0.16–2.96 | 0.623 | 0.74 | 0.18–3.02 | 0.672 |
| PD-L1 expression (Unknown vs. <1%) | 0.33 | 0.09–1.19 | 0.090 | 0.30 | 0.09–1.07 | 0.063 |
| Baseline NLR (per IQR) | 0.35 | 0.10–0.91 | 0.028 | 0.26 | 0.09–0.70 | 0.008 |
| Baseline LMR (per IQR) | 1.04 | 0.71–1.31 | 0.824 | 0.97 | 0.67–1.40 | 0.856 |
| Baseline PLR (per IQR) | 1.23 | 0.56–2.54 | 0.581 | 1.20 | 0.73–1.97 | 0.467 |

Notes: ORs are from multivariable Firth penalized logistic regression to reduce small-sample bias. sHRs are from multivariable Fine–Gray competing-risk regression with disease progression or death treated as competing events. All estimates and confidence intervals are exponentiated coefficients. Bold values indicate statistical significance (*p* < 0.05).

Abbreviations: AEC, absolute eosinophil count; CI, confidence interval; ICI, immune checkpoint inhibitor; IQR, interquartile range; LMR, lymphocyte-to-monocyte ratio; NLR, neutrophil-to-lymphocyte ratio; OR, odds ratio; PLR, platelet-to-lymphocyte ratio; sHR, subdistribution hazard ratio.
